# Microstructure and Mechanical Properties of Recycled Aggregate Concrete in Seawater Environment

**DOI:** 10.1155/2013/306714

**Published:** 2013-09-26

**Authors:** Pengjun Yue, Zhuoying Tan, Zhiying Guo

**Affiliations:** ^1^State Key Laboratory of High-Efficient Mining and Safety of Metal Mines (USTB), Ministry of Education, Beijing 100083, China; ^2^School of Civil and Environmental Engineering, University of Science and Technology Beijing, Beijing 100083, China; ^3^Nuclear Engineering Investigation Institute of Guangdong, Guangzhou 51080, China

## Abstract

This study aims to conduct research about the microstructure and basic properties of recycled aggregate concrete under seawater corrosion. Concrete specimens were fabricated and tested with different replacement percentages of 0%, 30%, and 60% after immersing in seawater for 4, 8, 12, and 16 months, respectively. The basic properties of recycled aggregate concrete (RAC) including the compressive strength, the elastic modulus, and chloride penetration depth were explicitly investigated. And the microstructure of recycled concrete aggregate (RCA) was revealed to find the seawater corrosion by using scanning electron microscope (SEM). The results showed that higher amount of the RCA means more porosity and less strength, which could lower both the compressive strength and resistance to chloride penetration. This research could be a guide in theoretical and numerical analysis for the design of RAC structures.

## 1. Introduction

Nowadays recycling of demolition concrete is beneficial and necessary from the viewpoint of environmental preservation and effective utilization of resources. In order to realize this, it is necessary to use demolition concrete as recycled aggregate for new concrete. To make it work, considerable amount of research has been conducted. Although some mechanical properties of recycled concrete may be generally lower than those of normal concrete, they are still sufficient for practical application in constructions [1–3]. 

For the key mechanical properties of RAC which are the compressive strength, elastic modulus, the bond strength, and tensile and the flexural strengths of such concrete, researchers have paid more and more attention to it globally. Rühl and Atkinson [4] reached a conclusion that the peak strain increases as the recycled aggregates increase after they investigated the complete stress-strain curve of recycled concrete with different replacement content. López-Gayarre et al. [5] discovered that modulus of elasticity of recycled aggregate concrete was slightly lower than that of conventional concrete when the replacement was less than 50% by weight. Kou et al. [6] and Olorunsogo and Padayachee [7] found that increasing of recycled aggregate as coarse aggregate in a concrete mixture resulted in higher oxygen permeability and chloride ion penetration than those of conventional concrete. Adom-Asamoah and Afrifa [8] discovered that the trends in the development of compressive and bending strengths of plain phyllite concrete were similar to those conventional concrete, but the compressive and bending strengths of phyllite concrete mixes were on the average 15–20% lower than those of the corresponding granite concrete mixes. Somna et al. [9] learned that the modulus of elasticity of recycled aggregate concrete with and without ground bagasse ash was lower than that of conventional concrete by approximately 19% after their investigation. Tangchirapat et al. [10] used ground palm oil fuel ash (POFA) with high fineness to improve the mechanical properties and durability of concrete and learned that ground POFA could improve the compressive strength and reduce the water permeability of recycled aggregate concretes. Chen [11] conducted the research of influences of different renewable aggregate ratios on the basic recycled concrete (RC) replacement mechanical properties and found that RC mechanical properties decrease with renewable aggregate replacement rate increasing. Xiao et al. [12] found that there exist no obvious differences of compressive fatigue behavior between RAC and natural aggregate concrete after their investigation. Lu et al. [13] conducted the experiment of recycled aggregate concrete (RAC) specimens prepared with five different amounts of recycled coarse aggregate (i.e., 0, 25%, 50%, 75%, and 100%) subjected to impact loading based on split Hopkinson pressure bar tests and found that the impact properties of RAC exhibit strong strain-rate dependency and increase approximately linearly with strain-rate, and the transition point from low strain-rate sensitivity to high sensitivity decreases with the increase of matrix strength.

However, all these researches were mainly focusing on the properties of RAC in normal condition, and rare was reported about properties of RAC with seawater corrosion, which prohibits a wider application of recycled concrete in the practical design of engineering structures. As mankind is stepping forward to explore the ocean, huge demands of construction materials are in need to execute the project. As said before, RAC was a feasible and green material in modern society. So it is of great necessity to learn the properties of RAC under seawater corrosion, which will be conducive to the application of RAC in marine environment.

In this paper, experiments were conducted to provide a comprehensive analytical evaluation of basic properties of RAC with seawater corrosion. Test results were compared with the normal concrete under the same conditions; the differences of compressive strength and elastic modulus were explicitly analyzed, and the chloride penetration depths and the microstructure of RCA were also investigated, by which the mechanism of seawater corrosion was discussed and revealed explicitly. The results obtained in this paper are significant to efficiently use recycled concrete in practical engineering especially in marine environment.

## 2. Experimental Descriptions

### 2.1. Materials and Mix Proportions

Ordinary Portland cement and river sand with fineness modulus of 2.8 were used in this study. The natural coarse aggregate (NCA) and recycled coarse aggregate (RCA) were obtained from a building demolition in Pudong Avenue, Shanghai, China. Their physical properties are shown in Table 1.

The mix proportion was C25 mixing methods as the national code of China [14] specified (C25 is the standard value of 28-day compressive strength 25 MPa), which was also suggested by Ye et al. [15]. And the ration of water to cement ration (W/C) was kept constant at 0.55. The mixture included three groups, and their main difference lied in the replacement percentage, which was 0%, 30%, and 60%, respectively. The mix proportions of concretes are shown in Table 2.

### 2.2. Specimens Preparation

The preparations of specimens were performed in the Laboratory for Concrete Material Research at Shanghai Maritime University in Shanghai, China. All mixtures were conducted under laboratory conditions. 

To simulate the seawater corrosion, seawater was picked up from the Huang Hai Sea, which is one of the four seas of China. Its content is listed in Table 3, which had been reported by Ge [16]. 

To obtain the compressive strength, 36 specimens are submerged in the seawater for 8 hours and out for 16 hours every day after 28-day curing, which is to simulate seawater corrosion in marine environment. The duration of this dry-wet circulation lasted for 4, 8, 12, and 16 months, respectively. And each group to be tested included three specimens in the same condition. And the loading setup was microcomputer controlled electrohydraulic servo tester.

To get the chloride penetration depth, specimens in 100 × 100 × 100 mm cubes were cut at the mid-height to obtain two pieces of concrete samples after 28-day curing. Nonshrinkage epoxy resin was cast around the surface of the 24 concrete samples prepared for determination of the chloride penetration depths to control chloride ions diffused into concrete along one dimension. Then the concrete samples were immersed in seawater. After immersing in seawater for periods of 4, 8, 10, and 12 months, the chloride penetration depths were measured, respectively, by using the methods suggested by Otsuki et al. [17].

## 3. Results and Discussion

### 3.1. Test Results and Validation

After the test, the compressive strength was obtained by following equation:

(1) $$\begin{array}{c} \sigma_{i}=\frac{F}{A}, \end{array}$$

where *σ*_*i*_ is the compressive strength (MPa); *F* represents the peak value of the force (N); *A* is the area of compression zone in specimen (mm^2^). 

The test results of compressive strength are listed in Table 4.

**Figure 1:**
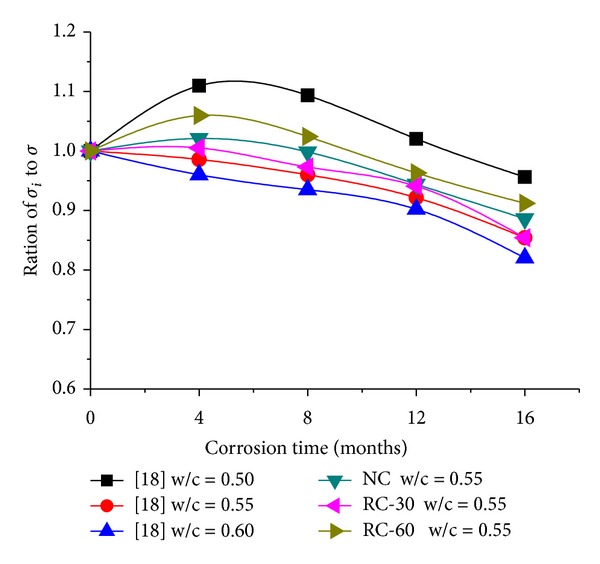
Dimensions of compressive strength of RAC with previous research.

 To check the accuracy of the test results, Figure 1 was drawn to make the comparison with previous research of normal concrete with the same seawater corrosion (*σ* is the standard value of 28-day compressive strength and it is 25 MPa).

Figure 1 shows that the curves of the RAC were similar to those of normal concrete which were conducted by Zhang and Wang [18]. This result demonstrates that the destruction mechanism is similar to that of normal concrete, which also confirms the validity of the tests. In addition, the compressive strength of RAC was bigger than standard value 25 MPa at the beginning of the test, but it turned to be smaller after the corrosion time exceeds 8 months. This may be interpreted that the compactness of RAC was gradually destroyed by the seawater penetration.

### 3.2. Compressive Strength

**Figure 2:**
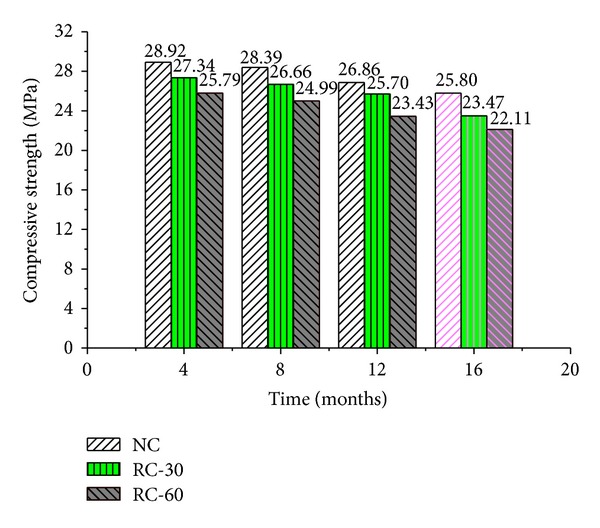
Compressive strengths of RAC with seawater corrosion.

The compressive strengths of three groups of RAC with seawater corrosion are shown in Figure 2.

Figure 2 indicates that the seawater had remarkable influences on compressive strength of RAC when replacement percentage and corrosion time increased. This also demonstrates that the plastic deformation and residual strength of RAC decrease and the destruction process accelerates with the increase of replacement percentage and corrosion time. 

 The compressive strength decreased by 1.16 to 2.31 MPa at different corrosion time when replacement percentage increased from 0% to 30%. And it decreased by 3. 13 to 3.69 MPa when replacement percentage increased from 0% to 60%. The decreasing range was nearly double when the replacement percentage exceeded 30%, which remind us that the compressive strength is obviously affected by the replacement percentage especially exceeding 30%. In Kasai's research [19], he suggested that replacement percentage should not exceed 30% in engineering application. From this research, it can be also concluded that the replacement percentage should also be less than 30% for the recycle aggregate concrete in seawater corrosion environment.

Another notable fact in Figure 2 is that compressive strength decreased with the corrosion time. The decreasing of RAC with seawater corrosion is presented in Table 5.

From Table 5, It can be seen that the compressive strength decreased at about 2% when seawater corrosion was within 8 months and the compressive strength degrades with a bigger decreasing range of 4% to 8% when the time exceeded 8 months. This indicates that the destruction process accelerated when internal bonding of concrete was gradually destroyed by the seawater. Therefore the corrosion of seawater costs time, and prevention measures should be taken in actual engineering application of RAC in marine environment.

### 3.3. Modulus of Elasticity

The elastic modulus *E*_*C*_ of the RAC was determined from compressive strength by the following empirical equation [10]:

(2) $$\begin{array}{c} E_{C}=5.639\sqrt{f_{c}}-4.952, \end{array}$$

where *E*_*C*_ represents the modulus of elasticity of concrete (GPa); *f*_*c*_ is the compressive strength of concrete (MPa). 

**Figure 3:**
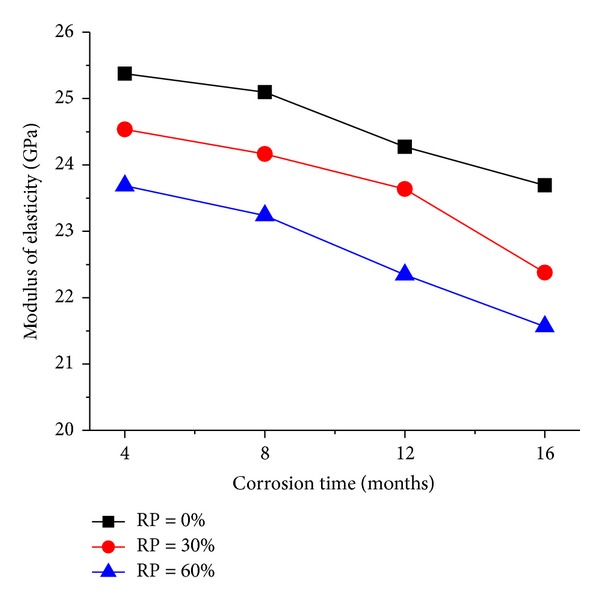
Elastic modulus of RAC with different corrosion time.

The elastic modulus of RAC is shown in Figure 3 (RP represents the replacement percentage). 

Figure 3 shows that the elastic modulus of RAC was lower than that of normal concrete (i.e., RP = 0%) with the same seawater corrosion, which was caused by the application of the RCA with a lower elastic modulus. And the decreasing range of elastic modulus was only about 7.5% when the replacement percentage varied; thus, it can be found that the elastic modulus changes slightly with the replacement percentage, which fits well with previous results by López-Gayarre et al. [5].

On the other hand, Figure 3 also shows the effects of corrosion time on the elastic modulus of the RAC. The elastic modulus dropped by 2% when the corrosion time was 8 months, and the elastic modulus was reduced by 9% when the corrosion time increased to 16 months. Therefore the decreasing trend accelerated when the corrosion time increased, which was because of the penetration of sea water into the internal of RAC. 

### 3.4. The Chloride Penetration Depth

**Figure 4:**
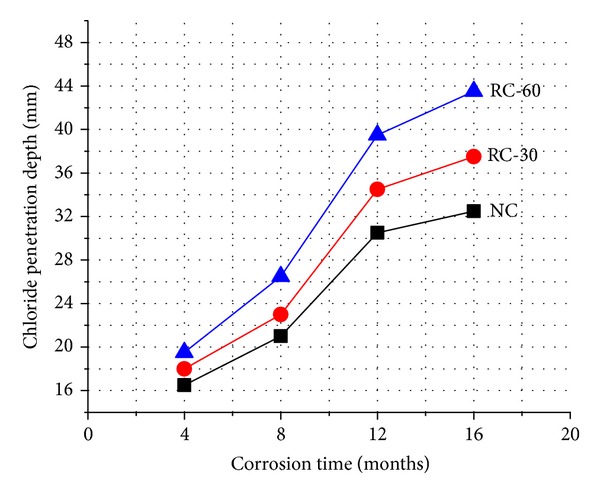
Chloride penetration depth of RAC.

The chloride penetration depth of RAC immersed in seawater is shown in Figure 4. 

The chloride penetration depths at the immersed time of 4, 8, 12, and 16 months of normal concrete were 16.5, 21.0, 30.5, and 32.0 mm, respectively, while those of RC-30 were 18.0, 23.0, 34.0, and 37.5 mm, respectively, and those of RC-60 were 19.5, 26.5, 39.5, and 43.5 mm, respectively. These results indicate that RAC had lower chloride resistance than that of normal concrete because the volume of pores in RAC was higher than that of normal concrete [9, 20]. This resulted from the attached mortar on the surface of RCA that had higher porosity than that of the crushed limestone. Moreover, the chloride ions could also penetrate through the interface between the attached mortar and old crushed aggregate [21]. 

From Figure 4, it can be also seen that the chloride penetration depth increased with the time for the same series, but the growth tended to be increasing at first and then decreasing. For instance, the chloride penetration depths of RC-30 were 18.0, 23.0, 34.0, and 37.5 mm, respectively. The growth was 5.0 and 9.0 mm when the corrosion time is from 4 to 8 months and from 8 to 12 months, respectively, but the growth was only 3.5 mm when the time increased from 12 months to 16 months. This is caused by the corrosion process occurring in the recycled aggregate concrete. The corrosion process may be slow at the first beginning of the seawater corrosion for the surface of concrete was well sealed. As the time went on, the chloride penetrated into the concrete and some of the concrete surface was destroyed, which may result in certain cracks forming. These cracks facilitated the penetration of the chloride so the corrosion process accelerated and growth reached its peak. However, for there are only limited numbers of ions participating in the corrosion process, the penetration of the chloride would slow down after the peak growth. So the increasing value became decreasing as the corrosion time increased from 8 to 16 months.

### 3.5. Microstructure of RCA

**Figure 5:**
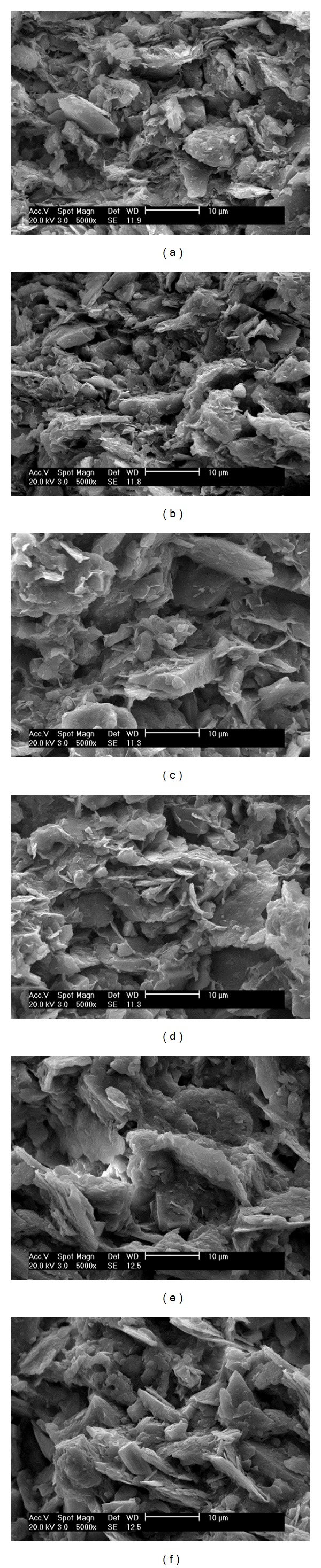
Microstructure of RAC with different replacement and corrosion time. (a) RP = 0% (before test). (b) RP = 0% (8 months corrosion). (c) RP = 30% (before test). (d) RP = 30% (8 months corrosion). (e) RP = 60% (before test). (f) RP = 60% (8 months corrosion).

In order to find the link between microstructure and the compressive strength, scanning electron microscope (SEM) was adopted to observe the internal structure of the RAC.  Figure 5 shows the microstructure of RAC before test and after 8 months seawater corrosion (RP represents the replacement percentage).

It can be found that the microstructure of RAC is obviously different with the replacement percentage and corrosion time. From Figures 5(a), 5(c), and 5(e), it can be found that the particle of the RCA was becoming thinner and tended to be more fragile to be destroyed as the replacement percentage increased. And the connection between the particles was shifting from the side-to-side link to face-to-face link, which would weaken the bonds of the particle, and resulted in the degrading of compressive strength. So the higher amount of the RCA means more porosity and less strength for RAC, which could lower the compressive strength. Therefore it can be concluded that the RCA should be well treated before engineering application and the suggested replacement percentage within 30% is right and feasible for actual engineering [19]. 

In addition, for every pair of (a) and (b), (c) and (d), and (e) and (f) in Figure 5, they all indicate that the RCA was degrading and destroyed by the seawater corrosion. The RCA tends to be more compact and in good connection before test; however, it shifts to be smaller particle and more broken bonding after 8 months seawater corrosion. Thus the corrosion of seawater on RCA is tremendous, which may have great impact on the compressive strength of RAC. Therefore the corrosion of seawater should not be underestimated and reinforced measures or preventions should be adopted in order to prolong the long-term bearing capacity of RAC in marine environment.

## 4. Conclusions

In this paper, test results for the properties of recycled aggregate concrete with seawater corrosion are presented and discussed. From this investigation, the conclusions can be drawn as follows.

1. The destruction mechanism of recycled aggregate concrete was similar to that of normal concrete in seawater corrosion environment, but the seawater had remarkable influences on properties of RAC in marine environment, and this influence became more obvious with the replacement percentage increased.
2. The compressive strength of RAC decreased with replacement percentage, and the decreasing range was nearly doubled when the replacement percentage exceeded 30%, so the suitable replacement percentage should not exceed 30% in seawater corrosion environment.
3. The compressive strength of RAC decreased with corrosion time. It decreased at 2% when the corrosion time was within 8 months, and it decreased at 4% to 8% when corrosion time exceeded 8 months.
4. The decreasing range of elastic modulus was at about 7.5% when the replacement percentage varied, but the effects of seawater on the elastic modulus of the RAC were more obvious with an increasing from 2% to 9%.
5. The recycled aggregate concrete had lower chloride resistance than that of normal concrete. The chloride penetration depth increased with the time for the same series, but the growth tended to be increasing at first and then decreasing.
6. The particle of the RCA was becoming thinner and tended to be more fragile to be destroyed as the replacement percentage increased. And the RCA tended to be more compact and in good connection before test; however, it shifted to be smaller particle and more broken bonding after 8 months seawater corrosion.

## Figures and Tables

**Table 1:** Physical properties of NCA and RCA.

Physical index	NCA	RCA
Grading (mm)	5–32.5	5–32.5
Bulk density (kg/m^3^)	1466	1305
Apparent density (kg/m^3^)	2812	2498
Water absorption (%)	0.45	9.15
Crush index (%)	4.12	14.9

**Table 2:** Mix proportions of concretes (kg/m^3^).

Number	Replacement percentage	Water/cement	Cement	Sand	NCA	RCA	Mixing water
NC	0	0.55	425	520	1305	—	234
RC-30	30	0.55	425	500	874	375	234
RC-60	60	0.55	425	480	496	745	234

**Table 3:** Content of seawater (g/L).

Content	NaCl	MgCl_2_	MgSO_4_	CaSO_4_	K_2_SO_4_	CaCO_3_
Amount	27.2	3.8	1.7	1.2	0.9	0.1

**Table 4:** Compressive strength of RAC with seawater corrosion (MPa).

Corrosion time (Months)	NC		RC-30		RC-60	
	Peak value	Mean value	Peak value	Mean value	Peak value	Mean value
4	28.90	28.92	25.30	27.34	25.40	25.79
	29.46		27.80		27.95	
	28.40		28.91		24.01	

8	31.30	28.39	25.58	26.66	26.87	24.99
	27.72		27.98		24.60	
	26.14		26.42		23.50	

12	26.20	26.86	25.40	25.70	23.20	23.43
	27.10		27.20		22.98	
	27.28		24.49		24.10	

16	27.80	25.80	23.98	23.49	22.64	22.11
	25.30		22.20		21.60	
	24.30		24.30		22.08	

**Table 5:** Decreasing of compressive strength with corrosion time.

Samples	Compressive strength (MPa) (%)		
	NC	RC-30	RC-60
4 months	28.92 (100)	27.34 (100)	25.79 (100)
8 months	28.39 (98)	26.66 (98)	24.99 (98)
12 months	26.86 (93)	25.70 (94)	23.43 (91)
16 months	25.80 (89)	23.49 (86)	22.11 (86)
